# Influence of drug transporters and stereoselectivity on the brain penetration of pioglitazone as a potential medicine against Alzheimer's disease

**DOI:** 10.1038/srep09000

**Published:** 2015-03-11

**Authors:** Kai Lun Chang, Hai Ning Pee, Shili Yang, Paul C. Ho

**Affiliations:** 1 Department of Pharmacy, Faculty of Science, National University of Singapore, Republic of Singapore; 2 Computational and Systems Medicine, Department of Surgery and Cancer, Faculty of Medicine, Imperial College London, London, United Kingdom

## Abstract

Pioglitazone is currently undergoing clinical trials for treatment of Alzheimer's disease (AD). However, poor brain penetration remains an obstacle to development of the drug for such intended clinical uses. In this study, we demonstrate that the inhibition of P-glycoprotein (P-gp) significantly increases brain penetration of pioglitazone, whereas inhibition of breast cancer resistance protein (BCRP) has little effect. We also investigate the stereoselectivity of pioglitazone uptake in the brain. When mice were dosed with racemic pioglitazone, the concentration of (+)-pioglitazone was 46.6% higher than that of (-)-pioglitazone in brain tissue and 67.7% lower than that of (-)-pioglitazone in plasma. Dosing mice with pure (+)-pioglitazone led to a 76% increase in brain exposure levels compared to those from an equivalent dose of racemic pioglitazone. Pure (+)-pioglitazone was also shown to have comparable amyloid-lowering capabilities to the racemic pioglitazone in an *in vitro* AD model. These results suggest that P-gp may act as a stereoselective barrier to prevent pioglitazone entry into the brain. Dosing with (+)-pioglitazone instead of the racemic mixture may result in higher levels of brain exposure to pioglitazone, thus potentially improving the development of pioglitazone treatment of AD.

Peroxisome proliferator-activated receptor-gamma (PPARγ) agonists such as rosiglitazone and pioglitazone have shown promising therapeutic potential against Alzheimer's disease (AD) in preclinical studies^1^,^2^,^3^,^4^,^5^. Both compounds have been suggested to have a role in regulating several aspects of AD, such as amyloid-β synthesis, inflammation, energy utilisation and lipid homeostasis^6^,^7^. Furthermore, these compounds play a role in restoration of mitochondrial activity^8^,^9^,^10^, offering the added potential of alleviating the mitochondrial dysfunction common in neurodegenerative diseases^11^,^12^.

The therapeutic potential of PPARγ agonists such as rosiglitazone has been well established in preclinical studies, although their efficacy has not yet been conclusively demonstrated in clinical trials^13^,^14^,^15^. Research interest in PPARγ agonists for treatment of AD was heightened after rosiglitazone demonstrated promising treatment effects in a preliminary clinical trial for AD patients^13^ and then performed well in a positive phase II clinical trial^14^. However, a subsequent phase III clinical trial failed to detect any evidence of rosiglitazone efficacy in the entire AD patient population enrolled in the study^15^. To account for this result, the authors hypothesised that effective levels of rosiglitazone may not be reaching the target tissues in the patient brain^15^. The authors proposed that the blockage may be related to the fact that rosiglitazone is a substrate of P-glycoprotein (P-gp), a major drug efflux transporter present at the blood-brain-barrier (BBB)^16^. P-gp expression at BBB is up-regulated in rat brain capillaries by neuroinflammation^17^, and the authors suggest that a similar condition in AD may further limit brain exposure to rosiglitazone and obviate its potential therapeutic benefits^15^. Accordingly, the authors suggest that other PPARγ agonists with higher brain penetration should be investigated.

Pioglitazone is the sole alternative PPARγ agonist available in the market; however, pioglitazone also demonstrates low brain penetration^18^. Similar to rosiglitazone, pioglitazone showed substantial preclinical promise for treatment of AD^2^,^5^,^19^. At present, three preliminary clinical trials for pioglitazone in AD patients have been reported, with contradictory outcomes^20^,^21^,^22^. A pilot clinical trial assessing pioglitazone's drug safety profile in an AD patient population did not detect clinical efficacy^20^. On the other hand, two preclinical trials performed by a research group in Japan reported positive treatment outcomes when AD patients with comorbid type II diabetes were given pioglitazone^21^,^22^. The discrepancies between the preclinical and clinical results of pioglitazone remain an obstacle to its development for the treatment of AD, resulting in a critical need for improved understanding of the factors determining pioglitazone efficacy. Such knowledge is especially necessary at present, as a new study from Takeda is currently recruiting 5800 subjects for a 5-year-long, phase III clinical trial to assess the therapeutic potential of pioglitazone in AD^23^.

Given the structural similarity of rosiglitazone and pioglitazone, we hypothesised that drug efflux transporters present at the BBB also limit brain penetration of pioglitazone. At present, no study has successfully addressed this factor. Therefore, in this study, we investigated the two most relevant drug efflux transporters, P-gp and breast cancer resistance protein (BCRP)^24^, as potential barriers to brain penetration of pioglitazone. Our *in vivo* experiments demonstrate that inhibition of P-gp leads to a significant although not substantial increase in the levels of pioglitazone in brain tissue, indicating the involvement of P-gp in limiting pioglitazone brain penetration. Because P-gp had previously shown clinically relevant stereoselectivity in its activity^25^,^26^, we further investigated the two stereoisomers of pioglitazone to explore stereoselectivity of brain uptake. Our findings show higher levels of (+)-pioglitazone in brain tissue than (-)-pioglitazone after a single dose of racemic pioglitazone in mice. We concluded that administration of purified (+)-pioglitazone rather than a racemic mixture may significantly increase brain tissue exposure in mice, and therefore represents a feasible strategy for overcoming low brain penetration of pioglitazone on a clinical level. Additionally, we demonstrated that (+)-pioglitazone has amyloid-lowering capabilities in an AD *in vitro* model comparable to those of the racemic pioglitazone. Thus, higher drug exposure of (+)-pioglitazone in the brain may improve clinical efficacy of the drug. Our findings represent an original contribution to the development of pioglitazone as a therapeutic treatment of AD and will be useful to research scientists and clinicians involved in pioglitazone phase III clinical trials for AD patients.

## Results

### Quantitative measurement of pioglitazone in biological samples using UPLC-MS/MS

A UPLC-MS/MS method was developed to quantify pioglitazone in both sample conditions (plasma and brain tissue). Multiple-reaction-monitoring (MRM) analyses were carried out using optimised *m/z* transitions for both pioglitazone (357 to 134) and rosiglitazone (358 to 135). The optimised operating conditions for mass spectrometry were: curtain gas, 15 psi (nitrogen); turbo gas temperature, 550°C; ion spray voltage, 5,500 V; nebulising gas, 40 psi; turbo gas, 40 psi; declustering potential, 54 V; entrance potential, 9 V; collision energy, 40 eV; collision cell exit potential, 4 V. The optimised retention times (RT) for pioglitazone and rosiglitazone were determined to be 0.72 minutes and 0.61 minutes, respectively. Five calibration samples were used to build calibration curves for both plasma and brain-tissue samples. Calibration curves were constructed from analyte-to-internal standard (IS) peak-area ratios. Weighted 1/x quadratic regression produced linear plots for plasma and brain samples over concentration ranges of 500–4000 ng/mL (*r*^2^ = 0.9995) and 50–3000 ng/mL (*r*^2^ = 0.9998), respectively. Pioglitazone concentrations for different samples types were then determined based on the respective calibration curves.

### *In vivo* investigation of P-gp and BCRP contributions to limited brain penetration of pioglitazone

**Figure 1:**
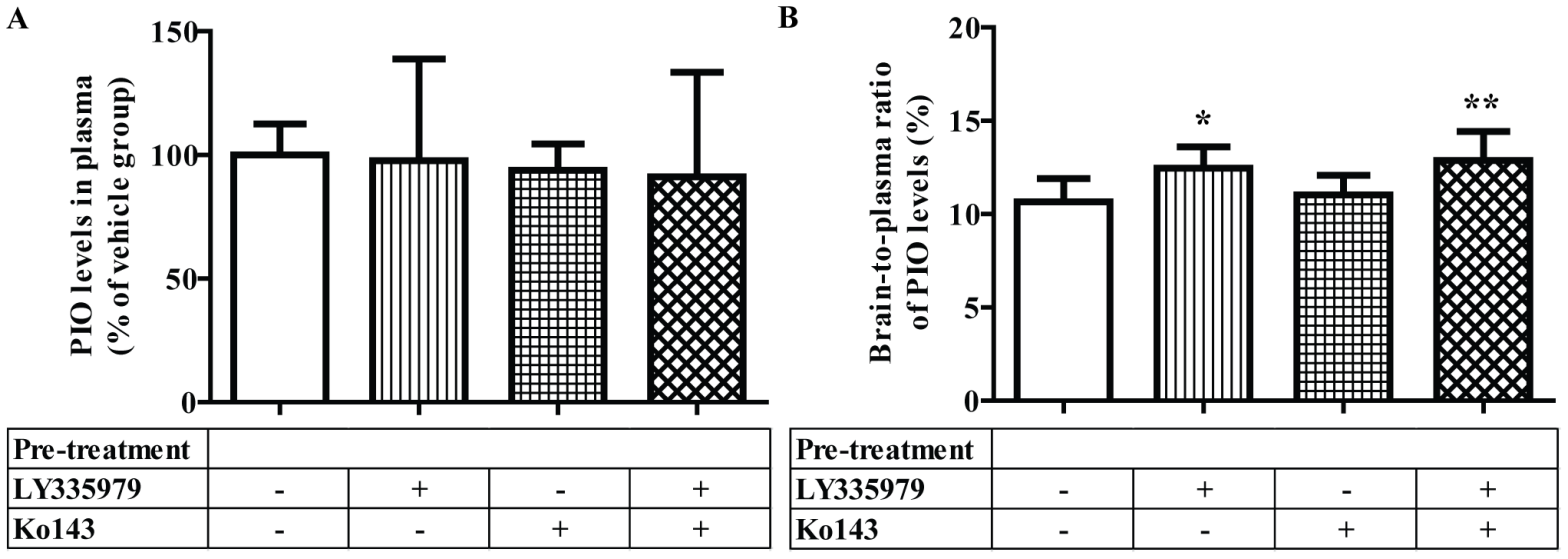
All animals were pre-treated with either the vehicle or the respective drug-transporter inhibitor (n = 6 for each treatment group) 30 minutes prior to drug treatment. Plasma and brain samples were then collected from the animals 30 minutes after pioglitazone treatment. (A) Pioglitazone levels in plasma samples harvested from mice given i.p. pioglitazone with or without pre-treatment of LY335979 (P-gp blocker) and/or Ko143 (BCRP blocker). (B) Brain-to-plasma ratios of pioglitazone concentrations calculated for each mouse given i.p. pioglitazone with or without pre-treatment of LY335979 and/or Ko143. Error bar represents one SD. * *P* = 0.0287 and ** *P* = 0.0235 when compared against vehicle-only pre-treatment group.

All animals were pre-treated with the respective drug transporter inhibitor(s) 30 minutes before administration of pioglitazone. Thirty minutes after treatment with pioglitazone, all animals were sacrificed and plasma and brain samples were collected. No significant differences were found in pioglitazone plasma concentrations among all four treatment groups (Figure 1A). The brain-to-plasma ratios of pioglitazone concentrations were used to assess penetration of brain tissue. Mice that were given vehicle as pre-treatments (the control group) had a pioglitazone brain-to-plasma concentration ratio of 10.65 ± 1.25%, which did not increase significantly when mice were pre-treated with Ko143 (BCRP inhibitor). However, pre-treatment with LY335979 (P-gp inhibitor) increased the pioglitazone brain-to-plasma concentration ratio significantly—although not substantially—by 16.92%, reaching 12.45 ± 1.15% (*P* = 0.0287), while pre-treatment with combined LY335979 + Ko143 also increased this ratio by 20.82%, reaching 12.87 ± 1.56% (*P* = 0.0235). No significant difference was observed between the brain-to-plasma ratios of the latter two treatment groups. Data on brain-to-plasma ratios are summarised in Figure 1B.

### Investigation of stereoselectivity of pioglitazone brain penetration using chiral HPLC-MS/MS

**Figure 2:**
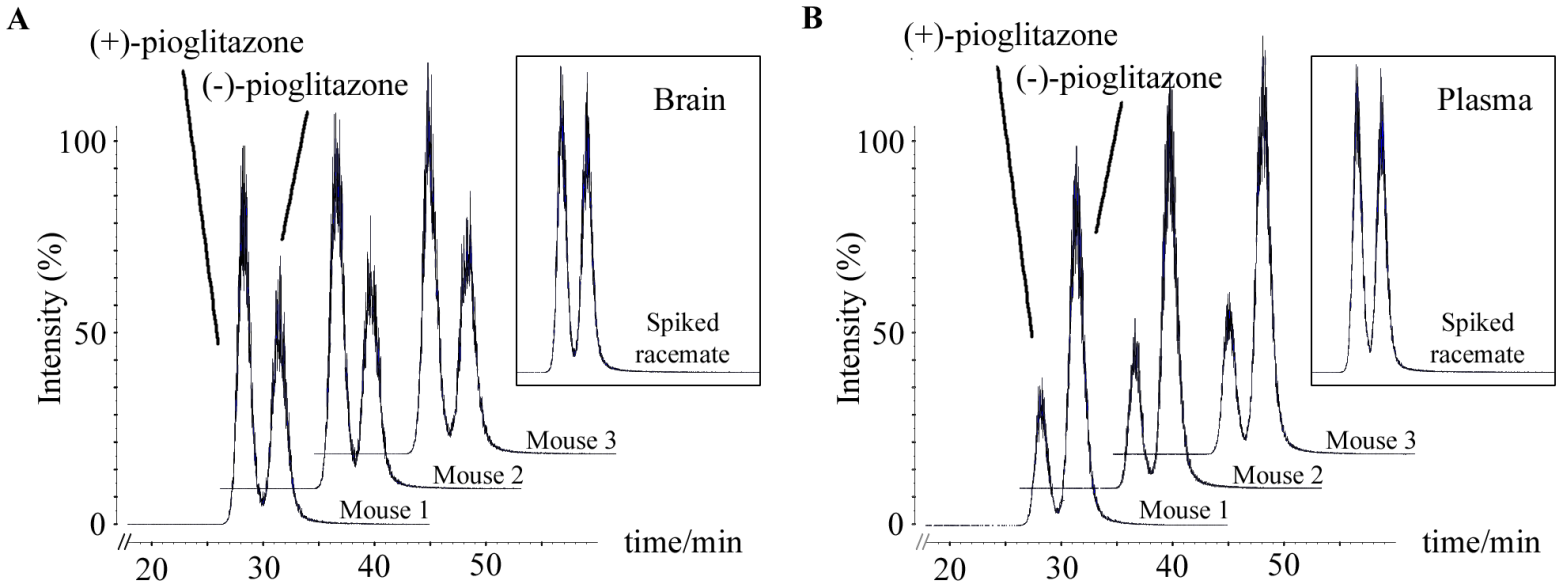
(A) Chiral separation of (+)-pioglitazone and (-)-pioglitazone in brain samples of mice administered with racemic pioglitazone; chromatogram in box shows (+)-pioglitazone and (-)-pioglitazone in blank brain sample spiked with racemic pioglitazone.(B) Chiral separation of (+)-pioglitazone and (-)-pioglitazone in plasma samples of mice administered with racemic pioglitazone; chromatogram in box shows (+)-pioglitazone and (-)-pioglitazone in blank plasma sample spiked with racemic pioglitazone.

Both (+)-pioglitazone and (-)-pioglitazone were successfully separated using an analytical reversed-phase chiral HPLC column (Chiralcel OD-R 10 μm particle size, 4.6 mm (i.d.) × 250 mm) with retention times (RTs) of approximately 28.00 minutes and 31.50 minutes, respectively. Chromatograms for both brain-tissue and plasma samples from all three mice are shown in Figure 2. The (+)-pioglitazone levels were consistently 46.6% higher than those of (-)-pioglitazone in brain-tissue samples. The opposite trend was observed in plasma samples; (+)-pioglitazone levels were consistently 67.7% lower than those of (-)-pioglitazone. No difference was found between the amount of (+)-pioglitazone and (-)-pioglitazone in control brain and plasma samples spiked with racemic pioglitazone.

### Purification and identification of (+)-pioglitazone

(+)-pioglitazone was successfully fractionated from the racemic mixture using semi-preparative HPLC. Purity was established by analysing three separate samples taken from the purified (+)-pioglitazone powder, which all showed >98.0% purity (n = 3, 98.08%, 98.12%, and 98.94%). The identity of (+)-pioglitazone was confirmed by measuring the optical activity of the purified powder dissolved in a solution of 90% acetonitrile and 10% isopropanol, supplemented with 0.1% acetic acid. Solvent alone was used as a zero reference before optical activity was measured for ten separately prepared (+)-pioglitazone solutions. Specific rotation in degrees ([α]_λ_^T^) for 1.11 mg/mL (+)-pioglitazone solution was calculated to be +0.8198 cm^2^/g, which confirmed the identity of purified powder to be the (+)-pioglitazone stereoisomer.

### *In vivo* comparative studies of racemic and (+)-pioglitazone drug-distribution profiles

**Figure 3:**
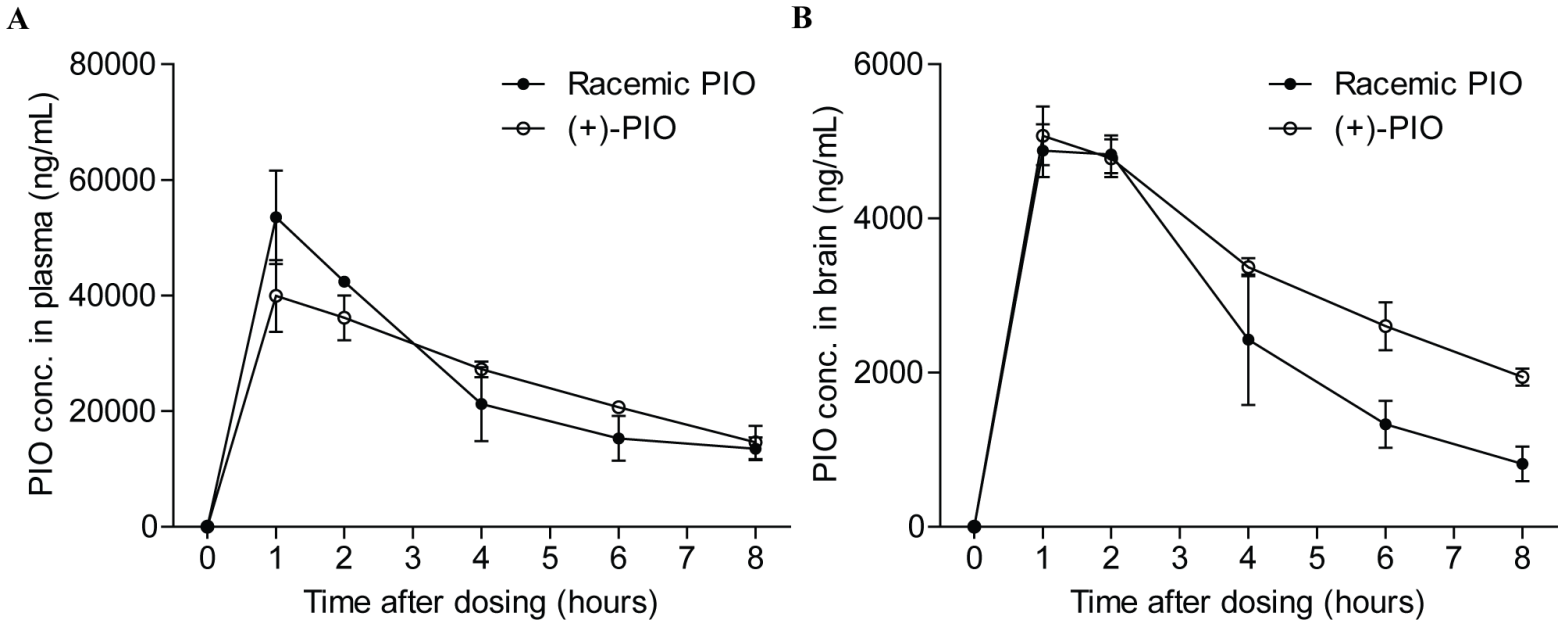
(A) Pioglitazone concentrations *versus* time plot for plasma samples harvested from mice given either racemic pioglitazone (AUC_0–∞_ = 2.62 × 10^5^ μg·hour/L) or purified (+)-pioglitazone (AUC_0–∞_ = 2.74 × 10^5^ μg·hour/L).(B) Pioglitazone concentrations *versus* time plot for brain samples harvested from mice given either racemic pioglitazone (AUC_0–∞_ = 2.27 × 10^4^ μg·hour/L) or purified (+)-pioglitazone (AUC_0–∞_ = 4.00 × 10^4^ μg·hour/L). Error bar represents one SD.

Our established UPLC-MS/MS method was used to quantify pioglitazone concentrations in plasma and brain samples harvested at different time points (1, 2, 4, 6, and 8 hours) after mice were given either racemic pioglitazone or (+)-pioglitazone. The profiles of mean plasma concentrations or mean brain concentrations of pioglitazone versus time are shown in Figure 3A and Figure 3B, respectively. Mice treated with racemic pioglitazone showed a significantly higher plasma concentration maximum (*C*_max_, 53.53 ± 8.07 μg/mL) than did mice treated with (+)-pioglitazone (39.92 ± 6.20 μg/mL, *P* = 0.0442). The peak time (T_max_) of plasma pioglitazone for mice from both treatment groups was 1 hour after drug administration. The area-under-the-curve (AUC)_0–∞_ values of plasma pioglitazone were 2.62 × 10^5^ μg·hour/L and 2.74 × 10^5^ μg·hour/L for mice that received racemic and (+)-pioglitazone, respectively. In contrast, the brain *C*_max_ was not significantly different between mice that received the racemic pioglitazone (4.88 ± 0.34 μg/mL) or the (+)-pioglitazone (5.07 ± 0.38 μg/mL). However, in mice that received (+)-pioglitazone, the brain pioglitazone concentration was significantly higher at 4, 6 and 8 hours after drug administration than mice treated with racemic pioglitazone, suggesting slower clearance of the (+)-isomer from the brain. The brain AUC_0–∞_ for mice that received (+)-pioglitazone (4.00 × 10^4^ μg·hour/L) was also remarkably higher (76%) than that for mice treated with racemic pioglitazone (2.27 × 10^4^ μg·hour/L).

### Relative amyloid-lowering capabilities of (+)-pioglitazone and racemic pioglitazone in CHO-APP_695_

**Figure 4:**
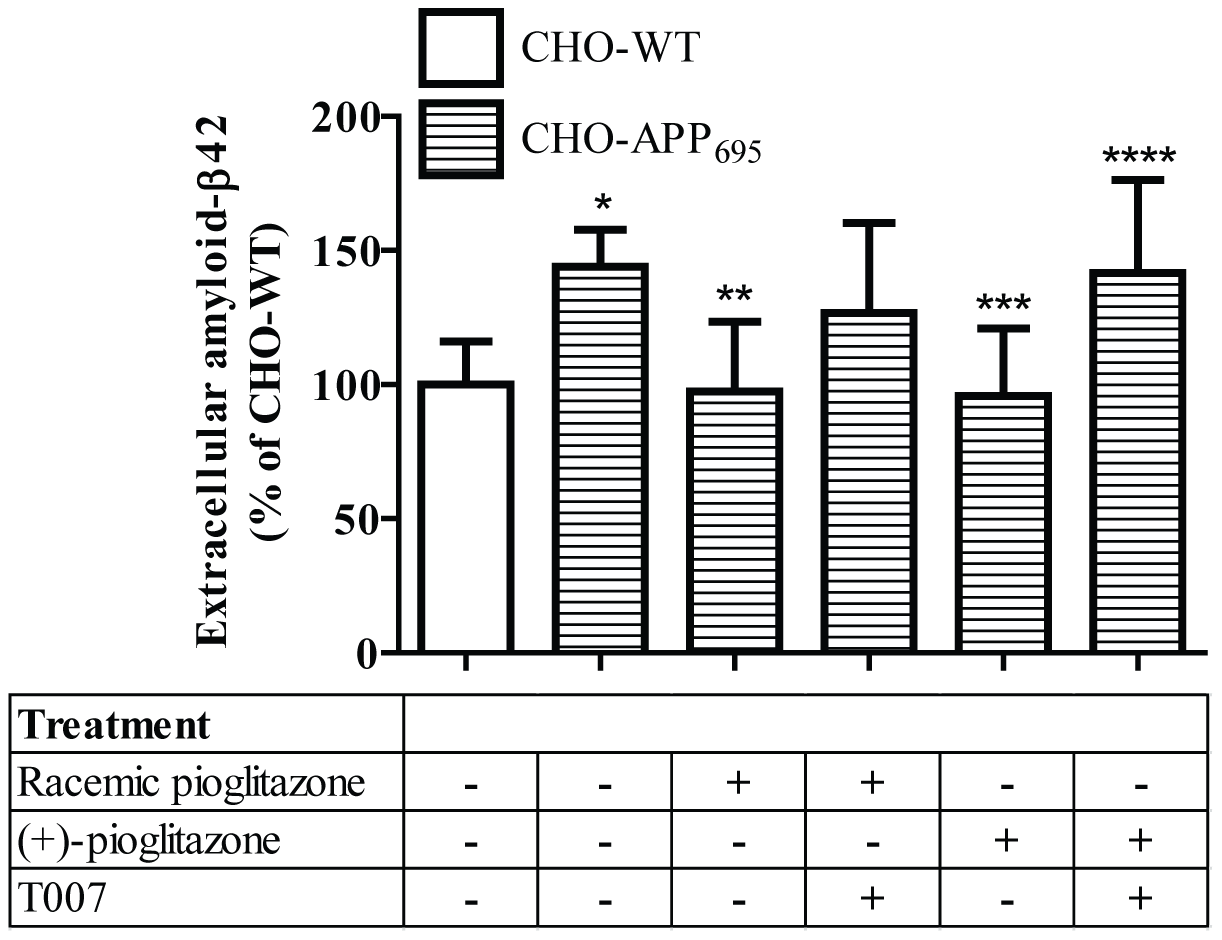
Extracellular amyloid-β42 levels in culture media samples harvested from both CHO-WT and treated or non-treated CHO-APP_695_ following 48 hours of post-seeding incubation. Error bar represents one SD. * *P* = 0.0002 when compared against CHO-WT, ** *P* = 0.0025 when compared against non-treated CHO-APP_695_, *** *P* = 0.0016 when compared against non-treated CHO-APP_695_, **** *P* = 0.0206 when compared against CHO-WT.

Forty-eight hours after seeding incubation, extracellular amyloid-β42 levels were increased significantly in CHO-APP_695_ (+43.8%) compared to CHO-WT cells. This increase in extracellular amyloid-β42 was reduced significantly by treatment with either the racemic pioglitazone or (+)-pioglitazone (*P* = 0.0025 and *P* = 0.0016, respectively, when compared with the non-treated CHO-APP_695_). The treatment effect of (+)-pioglitazone was nullified by co-administration of PPARγ selective blocker (T007), and a similar extent of nullification was observed with treatment of the racemic pioglitazone, although this treatment reversal effect did not reach the significant threshold. All data are summarised in Figure 4.

## Discussion

The main aim of this study is the exploration of factors that may limit brain penetration of pioglitazone, which is currently being investigated in clinical trials as a therapeutic agent for neurodegenerative diseases. Specifically, we focused our efforts on effects of drug efflux transporters at the BBB. AD transgenic mouse models are not ideal for our *in vivo* experiments because the BBB is compromised in AD transgenic mice^27^. An earlier clinical study indicated that no generalised abnormality existed in the BBB in AD patients^28^. A more recent study also indicated no evidence for additional BBB P-gp dysfunction in AD patients with microbleeds^29^. Therefore, in order to simulate the BBB of AD patients, it was appropriate to employ a mouse model with an intact BBB to investigate drug distribution in the brain for the objective to treat AD. For these reasons, healthy C57BL/6 mice were used as models of choice in our pioglitazone brain/plasma distribution study.

In this study, we observed significant, although not substantial increases in brain penetration of pioglitazone when mice were pre-treated with P-gp inhibitor (LY335979) or a combination of P-gp and BCRP inhibitors (Ko143). However, pre-treatment with BCRP inhibitor alone did not improve the brain penetration of pioglitazone. This shows that BCRP inhibition alone has no effect on brain penetration of pioglitazone at the dose used in this study; however, it may have synergistic effects when functioning in concordance with P-gp.

Mice that did not receive any drug-transporter inhibitor showed a brain-to-plasma pioglitazone ratio of 10.65%. To the best of our knowledge, this is the first study to investigate brain penetration of pioglitazone using C57BL/6 mice. Our observed brain penetration of pioglitazone is in agreement with previous studies using other animals, which have reported low pioglitazone brain penetration in general^18^,^30^. One study observed that cerebral spinal fluid (CSF) concentration of pioglitazone in monkeys was less than 3% of plasma concntrations^30^. Another study reported that approximately 18% of serum pioglitazone is found in the CSF of rats, dogs, and monkeys^18^.

We observed that brain penetration of pioglitazone increased upon pre-treatment with P-gp inhibitor or combined P-gp + BCRP inhibitors but not with BCRP inhibitor alone. This study is the first to investigate the contribution of P-gp and BCRP drug efflux transporters to the limitation of pioglitazone brain penetration. Our findings are comparable to another study that investigated the involvement of transporters at the human placental apical membrane on the penetration of rosiglitazone, a close structural analogue of pioglitazone. Having investigated effects of three drug efflux transporters (P-gp, BCRP and Multidrug Resistance Protein, MRP1) at the human placental apical membrane, Weiss et al. also found that rosiglitazone was predominantly transported by P-gp^24^. We noted a small rise from 16.92 to 20.82% in the brain-plasma ratios after treatment with the P-gp inhibitor or P-gp + BCRP inhibitors. Many possible reasons exist for the small observed increases in brain-plasma ratios after pre-treatment with the transporter inhibitors. One possible reason is that we only measured one time-point of the brain and plasma concentrations at 30 minutes after pioglitazone administration and calculated the corresponding brain-plasma concentration ratio. At that particular time-point, the effect of P-gp inhibition on the brain-plasma distribution may not be very evident. This postulation is supported by the findings regarding the stereoselective distribution of the drug in Figure 3, which show that the difference in the brain concentration after administration of the racemic and (+) pioglitazone is only observed after the peak concentration (C_max_) between 1–2 hours, i.e., after the absorption phase. This means that the stereoisomers may behave differently in the elimination phase than in the absorption phase, when it is cleared from the brain. P-gp is known to be stereoselective in activity^25^,^26^. It is possible that if the brain-plasma ratios had been obtained after the C_max_, the P-gp inhibition may show a greater influence on the ratios. However, our research objective in this preliminary experiment was simply to investigate whether pioglitazone is a substrate of P-gp and/or BCRP and not the extent of inhibition by P-gp.

Our findings clearly indicate that pioglitazone is a substrate of P-gp, which may contribute to the low brain exposure of pioglitazone observed in this and in other studies^18^,^30^. The low brain drug exposure is possibly an obstacle to the development of pioglitazone as a treatment of AD considering the fact that rosiglitazone's failure to reach therapeutic levels in brain tissue and thus its phase III AD trial failure was attributed to interactions with P-gp^15^. Co-administration of pioglitazone with P-gp regulators is a potential dosing strategy to enhance brain penetration and therapeutic effects of pioglitazone for treating AD. However, this dosing strategy is severely limited for a chronic disease such as AD by the poor solubility and nonspecific toxicity of even third-generation P-gp inhibitors^31^. In our attempt to devise a better strategy for enhancing pioglitazone brain penetration, we focused on the stereoselectivity of pioglitazone brain partitioning instead.

Remarkably, (+)-pioglitazone levels were clearly higher than (-)-pioglitazone levels in the brains of mice dosed with racemic pioglitazone, and the opposite trend was observed in the plasma. This result offers a potentially superior AD treatment strategy that uses (+)-pioglitazone, as this stereoisomer's higher presence in the brain suggests that more (+)-pioglitazone can be distributed to the brain and/or be retained for longer duration in the brain. Additionally, the lower presence of (+)-pioglitazone in plasma may translate to fewer undesirable drug effects or drug-induced toxicity in peripheral tissue organs. This combination of pharmacokinetic and target-delivery qualities makes (+)-pioglitazone a very promising candidate for development as a treatment of brain diseases such as AD.

Naturally, the next course of action is the determination of whether administration of purified (+)-pioglitazone can result in a higher presence of pioglitazone in the brain compared to the racemic form of pioglitazone currently available in the market. We hereby report for the first time that total pioglitazone exposure in brains of mice treated with (+)-pioglitazone is higher (+76%) than mice given racemic pioglitazone. Nonetheless, the total presence of pioglitazone in plasma of mice given (+)-pioglitazone was only 4% higher than that in mice fed with racemic pioglitazone. This result clearly demonstrates the brain-targeting property of (+)-pioglitazone, which offers two advantages to therapeutic application. First, increasing brain penetration of pioglitazone results in more drug compound crossing the BBB from the same dose given to the patient, thus therapeutic levels required for pioglitazone to exert its treatment effects in brain tissue are more easily reached. Second, the higher brain-targeting ability of (+)-pioglitazone allows a lower dose of pioglitazone to be given to AD patients to achieve the same therapeutic effect, thereby minimising undesirable drug effects or drug-induced toxicities in unintended organs. Our preliminary data also demonstrate that (+)-pioglitazone is comparable to racemic pioglitazone in its amyloid-lowering effect in an AD *in vitro* model (Figure 4). It should be noted that amyloid-lowering capacity is not the most directly relevant therapeutic effect of pioglitazone. The anti-inflammatory effect and restoration of mitochondrial dysfunction by pioglitazone are likely also involved in its potential therapeutic effects against AD^6^,^9^,^10^. However, amyloid-β disposition is one of the most established downstream biomarkers in AD pathology, and CHO-APP_695_ is an established amyloidogenic cellular model^10^. Thus, it is convenient and logical, although not certain, to use amyloid-lowering capacity to gauge the therapeutic potential of racemic mixture and the (+)-isomer using this *in vitro* model.

In conclusion, this study offers several novel findings that are valuable in drug development of pioglitazone for AD therapy, and our report is especially timely because pioglitazone is currently being investigated in clinical trials for several neurodegenerative diseases^23^,^32^. We demonstrate the role of P-gp as a barrier to pioglitazone reaching the brain, which has been discussed as a critical reason for the failure of rosiglitazone in phase III AD clinical trials^15^. We successfully devised a novel strategy to tackle poor brain penetration of pioglitazone by dosing with purified (+)-pioglitazone. However, further characterisation is needed to pursue (+)-pioglitazone as a therapeutic molecule for AD; in particular, the clinical safety and efficacy profiles remain to be established. Nevertheless, based on the present findings, enhanced brain retention of pioglitazone through administration of the (+)-isomer improves the promise of pioglitazone as an effective drug candidate for treating AD.

## Methods

### Chemicals

Ko143 (a selective inhibitor of BCRP) was purchased from Enzo Life Sciences (Lausen, Switzerland). LY335979 (a selective inhibitor of P-gp) was a generous gift from Eli Lilly and Company. T0070907 (T007, specific inhibitor of PPARγ receptor) was purchased from Cayman Chemical Company (Ann Arbor, MI). Pioglitazone and rosiglitazone (internal standard, IS) were purchased from Cell Molecular Pharmaceutical R&D (Xi'an, China). All other reagents used were analytical grade.

### Animals

In total, 64 adult male C57BL/6 mice (10–12 weeks of age) with body weights ranging between 23 and 28 g were obtained from the Centre for Animal Resources (CARE), National University of Singapore. The mice were housed in groups (maximum of 5 mice per cage) under standard conditions of humidity, temperature and 12-h light/dark cycle with *ad libitum* access to food and water. All mice were maintained under constant conditions for 4 days prior to experiments. All animal experiments were conducted in accordance with Singapore National Advisory Committee on Laboratory Animal Research (NACLAR) guidelines and approved by NUS Institutional Animal Care and Use Committee (IACUC).

### *In vivo* investigations of P-gp and BCRP contributions to limiting brain penetration of pioglitazone

A total of 24 mice were divided into four treatment groups (n = 6), where three groups received pre-treatment of efflux pump inhibitors (P-gp inhibitor LY335979, BCRP inhibitor Ko143, and a combination of LY335979 + Ko143) and one group received vehicle pre-treatment as a control. All test compounds (pioglitazone, LY335979, and Ko143) were dissolved in Milli-Q water using 1% DMSO as co-solvent. Pre-treatments of LY335979 (25 mg/kg), Ko143 (5 mg/kg) or a combination of both were given in a single intra-peritoneal (i.p.) injection 30 minutes before pioglitazone administration (i.p. injection, 30 mg/kg). At these selected doses, both LY335979 and Ko143 exhibited 50% inhibition of P-gp^33^ and BCRP^34^, respectively, in previous studies using mice, thus allowing previous effects to be compared with those in this study. Following another 30 minutes after pioglitazone administration, all mice were sacrificed by CO_2_ euthanasia and their blood samples were collected via cardiac puncture into Eppendorf tubes supplemented with heparin. Transcardial perfusion with saline was then performed on the sacrificed mice to remove traces of blood from organs before the whole brain tissues were harvested. All biological samples were kept on ice immediately after collection. Blood samples were centrifuged at 4000 g for 10 minutes and plasma samples (supernatant) were then carefully transferred into clean Eppendorf tubes. Plasma and all tissue samples were stored at −80°C until analysis. Plasma pioglitazone concentrations and brain-to-plasma pioglitazone ratios in samples harvested from all four treatment groups were used to evaluate the effects of pre-treatments with drug efflux transporter inhibitor(s) on distribution of pioglitazone. Comparisons of data between groups were carried out using two-tailed independent t-test with Welch's correction, and statistical significance was defined as *P* < 0.05.

### Sample preparation for quantitative measurement of pioglitazone in biological samples

All samples were thawed and kept on ice. Brain-tissue samples were homogenised with Milli-Q water in a ratio of 1 part tissue mass to 1 part water. Plasma and brain homogenate were then diluted 10-fold and 3-fold with Milli-Q water, respectively. 95 μL of diluted sample was transferred into a clean Eppendorf tube, and rosiglitazone (2.5 ng in 5 μL of DMSO) was added as an internal standard (IS) to each sample. 300 μL of methanol was then added to each tube to initiate protein precipitation for extraction of the analyte. All mixtures were vortex-mixed at high speed for 5 minutes and centrifuged at 14000 g (20 minutes at 4°C) to pellet the precipitated protein and sample debris. Supernatants were then transferred into injection vials for subsequent analysis using ultra performance liquid chromatographic system coupled with tandem mass spectrometer (UPLC-MS/MS). For calibration standards, blank plasma and tissue homogenates (spiked with 100, 500, 1000, 2500, 5000, and 10000 ng/mL pioglitazone) were prepared in a similar manner and used to build a calibration curve for each sample type.

### Instrument operating conditions of UPLC-MS/MS and data processing

Liquid chromatographic separations were performed using the Waters Acquity UPLC system fitted with Acquity UPLC BEH C_18_ 1.7 μm, 2.1 (i.d.) × 50 mm column (Waters Corp., MA, USA). Temperatures of column and autosampler were maintained at 45°C and 10°C, respectively. The mobile phases consisted of 1% (v/v) formic acid in Milli-Q water (solvent A) and 1% (v/v) formic acid in acetonitrile (solvent B) delivered at 0.6 mL/minute. The elution conditions used were as follows: isocratic at 20% solvent B (0–0.2 minute), gradient of 20% to 90% solvent B (0.2–1.5 minute), isocratic at 95% solvent B (1.5–1.8 minute), isocratic at 0.1% solvent B (1.8–2.2 minute) and isocratic at 20% solvent B (2.2–2.5 minute).

API 3200 triple quadrupole mass spectrometer (AB SCIEX, MA, USA), operating in positive electrospray ionisation mode, was used for detection of pioglitazone and rosiglitazone (IS). Multiple-reaction-monitoring (MRM) method was used to measure both pioglitazone (target compound) and rosiglitazone (IS). The following MS operating conditions were optimised: curtain gas, turbo gas temperature, ion spray voltage, nebulising gas, turbo gas, declustering potential, entrance potential, collision energy, and collision cell exit potential. Integration of peak areas and data processing were carried out using Analyst version 1.4.2 software (AB SCIEX). Analyte/IS peak area ratios were used to construct calibration plots for different tissue sample types, which were then used to determine concentrations of pioglitazone in experimental samples.

### Exploratory investigation of stereoselectivity in pioglitazone brain penetration using chiral HPLC-MS/MS

Using the same approach described in the *in vivo* experiment, we investigated the differential distribution of (+)-pioglitazone and (-)-pioglitazone in brain and plasma of mice (n = 3) that received vehicle-only pre-treatment and racemic pioglitazone i.p. injection (30 mg/kg). Brain and plasma samples were harvested 30 minutes after administration of pioglitazone (i.p. injection, 30 mg/kg), in a similar manner to that described above. Stereoisomers of pioglitazone were separated by reversed phase chiral high performance liquid chromatographic (HPLC) column. Brain and plasma samples from mice, as well as blank brain and plasma samples spiked with racemic pioglitazone, were subjected to chiral HPLC separation and quantified using tandem MS. Chiral liquid chromatographic separations were performed using Agilent 1200 HPLC system (Agilent Technologies, CA, USA) fitted with Chiralcel OD-R 10 μm particle size, 4.6 mm (i.d.) × 250 mm analytical column (Chiral Technologies Europe, Illkirch-Graffenstaden, France). Temperatures of the column and autosampler were maintained at 25°C. A mobile phase consisting of 60% (v/v) methanol in MilliQ water was delivered at 1.0 mL/minute under the isocratic elution mode. Detection and quantitation of pioglitazone was carried out using API 3200 MS (AB SCIEX) according to tandem MS operating conditions described above. The ratios of both stereoisomers relative to one another were used to determine their relative distribution in each sample type.

### Purification and identification of (+)-pioglitazone from racemic mixture

Purification of (+)-pioglitazone was carried out using the HPLC system coupled with a UV detector and automated fraction collector. The system consisted of a Shimadzu LC-10AT pump, SCL-10A system controller and FRC-10A fraction collector (Shimadzu Corporation, Kyoto, Japan). The chiral column used was a Chiralpak IA 5 μm particle size, 10 mm (i.d.) × 250 mm semi-preparative column (Chiral Technologies Europe, Illkirch-Graffenstaden, France). For every round of collection, 250 μL of pioglitazone solution (5 mg/mL) was injected and separated using isocratic mobile phase elution (90% acetonitrile and 10% isopropanol, supplemented with 0.1% acetic acid), and detection wavelength was set at 266 nm to monitor analyte peak elution. HPLC elution flow rate was set to 2 mL/minute and temperature was maintained at 40°C. Racemic pioglitazone was dissolved in solvent of a similar composition to the mobile phase to prepare the solution for injections. The retention time (RT) for (+)-pioglitazone was approximately 15.00 minutes with peak width of 2 minutes. The RT of (-)-pioglitazone was 40.00 minutes, evidently separable from the (+)-isomer, therefore allowing collection of the latter isomer. The collected fraction was then dried using a rotary evaporator to obtain purified powder of (+)-pioglitazone. Purity of (+)-pioglitazone was then examined by re-constituting the powder in mobile phase solvent and analysing the re-constituted solution using the previously established chiral HPLC separation coupled with MS detection (API 3200 MS, AB SCIEX) using MRM method (m/z transitions of 357 to 134 for pioglitazone detection). The identity of purified (+)-pioglitazone was confirmed by measuring its optical activity using a DIP-1000 Spectropolarimeter (Jasco Inc., MD, USA). Optical rotation measurements were performed in pioglitazone solution (1.11 mg/mL) in a 10-mm cell and were measured at a wavelength of 589 nm and temperature of 26°C. Specific rotation (cm^2^/g) was calculated using the equation [α]_λ_^T^ = α/L * c (where [α]_λ_^T^ is the specific rotation, α is the observed rotations in degrees, L is the cell path in dm, and c is the sample concentration in g/100 mL).

### *In vivo* comparative studies of racemic and (+)-pioglitazone drug-distribution profiles

A total of forty mice were split into two treatment groups (n = 20), with one group receiving a single dose of the racemic pioglitazone (30 mg/kg, p.o.) and another group receiving the purified (+)-pioglitazone (30 mg/kg, p.o.). Both compounds were dissolved in Milli-Q water using 1% DMSO as co-solvent. Following oral administration of the test compounds, mice from both treatment groups were split again into five subgroups (n = 4), and mice belonging to different subgroups were sacrificed at different time points (1 hour, 2 hours, 4 hours, 6 hours, and 8 hours after oral dosing). All mice were sacrificed by CO_2_ euthanasia and blood samples were taken via cardiac puncture. Blood samples were then centrifuged at 4000 g (10 minutes) to collect the plasma. Whole brains were collected following transcardial perfusion with saline to remove blood from organs. Sample treatment for measurement of pioglitazone in plasma and brain samples using UPLC-MS/MS was similar to the procedure described above. Pioglitazone concentrations *versus* time graphs were constructed for both brain and plasma samples, and area under curve (AUC) for both (+)-pioglitazone and racemic pioglitazone were then used to assess each tissue's exposure to pioglitazone. AUC was calculated from time 0 hour to infinity (AUC_0–∞_) for both types of samples using the trapezoidal rule. Comparisons of pioglitazone concentrations in different sample matrices between the two treatment groups were performed using two-tailed independent t-test with Welch's correction, and statistical significance was defined as *P* < 0.05.

### Culture conditions for the CHO-APP_695_ cell line, an AD *in vitro* model

CHO (Chinese hamster ovary) cells stably transfected with mouse APP 695 (CHO-APP_695_) were used as the AD *in vitro* model in this experiment. CHO-APP_695_ and the wild type (CHO-WT) were kindly provided by G. S. Dawe (National University of Singapore, Singapore). Both cell lines were incubated at 37°C in the presence of 5% CO_2_ for all cell culture work. Cell lines were maintained in DMEM culture medium (Sigma D1152) containing 10% FBS and supplemented with 100 units/mL of penicillin and 100 μg/mL of streptomycin.

### Comparing efficacies of (+)-pioglitazone and racemic pioglitazone on lowering extracellular amyloid-β42

When cells were used for experiments, both CHO-APP_695_ and CHO-WT were standardised at third passage and harvested for seeding when the cells were at log-phase growth. Cells were seeded (n = 7) on 24-well cell culture plates at 1 × 10^5^ cells/well in 0.5 mL of culture medium. To evaluate amyloid-lowering effects of (+)-pioglitazone and racemic pioglitazone on extracellular amyloid-β42 levels, additional groups (n = 7) of CHO-APP_695_ treated with (+)-pioglitazone, (+)-pioglitazone + T007, racemic pioglitazone, and racemic pioglitazone + T007 were also seeded. A concentration of 10 μM was used for all treatment compounds because this concentration has been shown to have no significant impact on cellular proliferation rates of CHO-APP_695_ and CHO-WT when cultured under similar experimental conditions in our previous study^10^. Upon completion of seeding after 24 hours, the culture medium in each well was replaced with 0.5 mL of fresh medium. Seeded cells were incubated further for 48 hours before the culture medium was individually harvested. Harvested samples were briefly centrifuged (800 g) for 5 minutes at 4°C before supernatants were transferred into clean 1.8-mL cryogenic vials and snap frozen in liquid nitrogen. All harvested samples were stored at −80°C until analysis.

### Measurement of extracellular amyloid-β42

Amyloid-β42 levels in culture media were harvested 48 hours after seeding incubation and were quantitatively measured using colorimetric sandwich ELISA kits according to the protocols provided by the manufacturer (#SIG-38954 and #SIG-38956 from Covance, Princeton, NJ). Undiluted culture medium was used to detect amyloid-β42. Non-normalised data were then statistically compared using two-tailed independent t-test with Welch's correction and statistical significance was defined as *P* < 0.05. All absorbance readings were measured using a Tecan Infinite M200 microplate reader (Tecan, Switzerland).
